# Relationship between alcohol consumption and adverse childhood experiences in college students–A cross-sectional study

**DOI:** 10.3389/fpsyg.2022.1004651

**Published:** 2022-10-12

**Authors:** Karolína Šulejová, Dávid Líška, Erika Liptáková, Mária Szántová, Michal Patarák, Tomáš Koller, Ladislav Batalik, Michael Makara, Ľubomír Skladaný

**Affiliations:** ^1^2nd Department of Internal Medicine, Faculty of Medicine, HEGITO (Div Hepatology, Gastroenterology and Liver Transplant), F. D. Roosevelt Teaching Hospital, Slovak Medical University, Banská Bystrica, Slovakia; ^2^Faculty of Arts, Department of Physical Education and Sports, Matej Bel University, Banská Bystrica, Slovakia; ^3^Faculty of Economics, Technical University of Košice, Košice, Slovakia; ^4^3rd Department of Internal Medicine, Medical Faculty of Comenius University Bratislava, University Hospital, Bratislava, Slovakia; ^5^Psychiatric Clinic, F. D. Roosevelt Teaching Hospital, Slovak Medical University, Banská Bystrica, Slovakia; ^6^Gastroenterology and Hepatology Subdivision, 5th Department of Internal Medicine, Comenius University Faculty of Medicine, University Hospital Bratislava, Bratislava, Slovakia; ^7^Central Hospital of Southern Pest, Budapest, Hungary; ^8^Department of Rehabilitation, University Hospital Brno, Brno, Czechia; ^9^Department of Public Health, Faculty of Medicine, Masaryk University, Brno, Czechia; ^10^2nd Department of Internal Medicine, Faculty of Medicine, P. J. Safarik University, Košice, Slovakia

**Keywords:** adverse childhood experience, alcohol consumption, COVID-19 pandemic, negative impact pandemic, audit

## Abstract

**Background:**

Alcohol consumption is an important issue. Adverse childhood experiences (ACEs) can affect alcohol consumption later in life. Therefore, the main objective of this study was to test the association between ACE and the alcohol consumption in college students.

**Materials and methods:**

A cross-sectional study on college students was conducted during December 2021 and January 2022, Through the school web system, students received a standard questionnaire on alcohol consumption (AUDIT) and ACEs. The study involved 4,044 participants from three universities in Slovakia.

**Result:**

Compared to men, the incidence of emotional abuse by a parent, physical abuse by a parent, and sexual abuse was significantly higher in women (*p* < 0.001). Furthermore, women reported greater emotional and physical neglect (p < 0.001). The incidence of a high or very high AUDIT score in college students with ACE-0, ACE-1, ACE-2, ACE-3, and ACE-4+ was 3.8, 4.7, 4.1, 6.4, and 9.3%, respectively.

**Conclusion:**

More adverse childhood experiences were associated with increased alcohol consumption in both male and female university students. Baseline drinking was higher in male students, but increased drinking in relation to an increase in ACEs was higher in female students. These results point to gender-specific driving forces and targets for intervention.

## Introduction

Adverse childhood experiences, including several types of abuse (physical, sexual, and emotional abuse, neglect, being the witness of domestic violence, and other serious household dysfunctions), indicate potentially established traumatic sources of stress (Heim et al., 2002; DeLisi and Beauregard, 2018). Adverse Childhood Experiences (ACEs) can have lasting negative effects on health, wellbeing, education, and job potential (Herzog and Schmahl, 2018). Adverse childhood experiences are associated with poorer mental health and a higher risk of psychological and psychiatric problems (Kalmakis and Chandler, 2014; Reiser et al., 2014; Serafini et al., 2014). Several studies have suggested that ACEs are associated with a negative perception of physical health (Suliman et al., 2009; Duke et al., 2010; Putnam et al., 2013; Marackova et al., 2016; Scott and Semple, 2017; Lackova Rebicova et al., 2020). Furthermore, higher ACE scores increase the risk of developing several diseases in adulthood, increased alcohol consumption, and many types of addictions (Felitti, 2002; Chartier et al., 2010; Mouton et al., 2016).

With a lifetime prevalence of up to 14%, alcohol use disorder (AUD) is one of the main public health problems (Anda et al., 2002). In Slovakia, three out of four adults drink alcohol on occasion or regularly. Additionally, alcohol in Slovakia is more socially acceptable than other psychoactive substances, and the general societal environment can be considered alcogenic. Children who grew up in families with parental alcohol abuse are at increased risk of developing AUD with a wide range of psychological, physical, and socioeconomic health consequences (Koppes et al., 2006; Strine et al., 2012; Rehm and Shield, 2014; Shield et al., 2014; Frankenberger et al., 2015; Lee and Chen, 2017; Seitz et al., 2018). Identifying factors in all domains that are known to increase the risk of AUD is important for the development of more effective prevention and treatment strategies (Thompson et al., 2008). The prevalence of liver cirrhosis is highest in Slovakia, with alcohol-associated liver disease (ALD) being the most common aetiology (Sepanlou et al., 2020).

To better understand alcohol-related behaviour during, the Alcohol Use Disorder Identification Test (AUDIT) was administered to detect alcohol consumption in college students. Therefore, the main objective of this study was to test the association between adverse childhood experiences and the alcohol consumption of college students.

Therefore, our hypothesis is as the following:

$H0:\mu_{1}=\mu_{2}$ (there is no difference in the mean of ACE among alcohol non-users and users).

$H1:\mu_{1}\neq \mu_{2}$ (there is a difference in the mean of ACE among alcohol non-users and users).

## Materials and methods

This analytical cross-sectional study was conducted between December 2021 and January 2022. Three Slovak universities were included in the study: (1) Matej Bel University in Banská Bystrica, (2) the Technical University of Košice, and (and attendance at boarding schools increased the odds of alcohol 3) Slovak Medical University Bratislava, Faculty of Health Care in Banska Bystrica. After obtaining their informed consent, the students completed structured questionnaires. The main outcomes were ACEs and alcohol consumption by Alcohol Use Disorder Identification Test (AUDIT) (Ballester et al., 2021). The questionnaire was compiled using Google Forms and distributed through the universities’ online systems, and data were extracted into an Excel spreadsheet. College students self-reported responses. Demographic variables were obtained from students at the beginning of the questionnaire. The inclusion criteria were satisfied if the participants were college students at a Slovak university. The minimum required age was 18. Students with visual impairment were not included in the study. All procedures performed in studies involving human participants were in accordance with the ethical standards of the institutional and/or national research committee and with the 1964 Helsinki Declaration and its later amendments or comparable ethical standards. Informed consent was obtained from all individual participants involved in the study. The study was approved by the Ethics Committee of Matej Bel University under the number 2200/2021.

### Adverse childhood experience

The ACE questionnaire is an assessment tool that measures various types of abuse and other kinds of adverse experiences in childhood (Hardt and Rutter, 2004; Haaris Sheikh et al., 2017; Choi et al., 2020; McLennan et al., 2020). The ACE questionnaire includes 10 domains, each assigned zero (particular ACE not experienced) or one (ACE experienced) point and summarized as a final score on a scale of 0–10. The domains are physical, psychological, and sexual abuse, emotional and physical neglect, and domestic dysfunction, such as alcohol abuse, drug use at home, loss of a parent, mental illness, violent treatment of the mother, and imprisonment of the parent. Scores are expressed as the sum of points with a higher score representing more ACEs (Anda et al., 1999).

### Alcohol use disorder identification test

The AUDIT is a simple and effective method of detecting unhealthy alcohol use, defined as risky or dangerous consumption or any alcohol use disorder (Daeppen et al., 2000; Kuitunen-Paul and Roerecke, 2018; Verhoog et al., 2020; Ballester et al., 2021). AUDIT can also help identify alcohol dependence and the specific consequences of harmful drinking. The Slovak standardized version was used. The final AUDIT score is divided into low (scores between 0 and 7), medium (scores between 8 and 15), high (scores between 16 and 19), and very high (scores 20 and above). The questions addressed: (1) frequency of drinking, (2) amount of alcohol ingested, (3) symptoms of addiction (impaired control of drinking, increased importance of drinking, and early morning drinking), and (4) surrogates of harmful alcohol use (guilt after drinking and alcohol-related injuries).

### Participants

The study involved 4,044 students from the three universities in Slovakia: Technical University of Košice, Matej Bel University in Banská Bystrica, and the Slovak Medical University Bratislava, Faculty of Healthcare in Banská Bystrica. The characteristics of the cohort are shown in Table 1. The questionnaires were collected using Google Forms.

**Table 1:** Participants’ characteristics.

		Total (*N* = 4,044)
Age (years)	Mean	22.33 (SD = 4.8)
Gender	Women	1,850 (45.7%)
	Men	2,194 (54.3%)
School year	First year	1,342 (33.2%)
	Second year	951 (23.5%)
	Third year	745 (18.4%)
	Fourth year	530 (13.1%)
	Fifth year	421 (10.4%)
	Sixth year	55 (1.4%)
Type of study	Full-time	3,697 (91.4%)
	Remote	347 (8.6%)
University	Technical University	2,769 (68.5%)
	Matej Bel University	1,066 (26.4%)
	Slovak Medical University	209 (5.2%)

### Statistical analysis

The results were uploaded in an Excel spreadsheet and subsequently subjected to a statistical analysis using the IBM SPSS Statistics 19 software. A two-proportion z-test, pooled for H0: *p*1 = *p*2, was used to test the equality of proportion between the groups of men and women. The absolute risk calculation was used to express the relationship between the final ACE score and the AUDIT score (absolute risk = the number of events in the treated group divided by the number of people in that group). As the distribution of ACE scores is not normal, the nonparametric Mann–Whitney U test was used to test the difference in mean score of ACE between group of alcohol users and group of alcohol non-users.

The sample size for this study was calculated using the following formula:


$$n=\frac{\frac{Z^{2}*p*q}{e^{2}}}{1+\frac{Z^{2}*p*q}{e^{2}*N}}$$

where *n* = sample size, *N* = population size, *Z* = *Z* score, *p* = probability of success, *q* = probability of failure, and *e* = margin of error. The confidence level was set at 95%, the margin of error at 5%, the probability of success at 50%, and the population of Slovak university students at 133,558. Therefore, a sample of 196 subjects was required.

The internal consistency of the AUDIT and ACE scores was measured separately by the Cronbach’s alpha coefficient of reliability. The AUDIT had a Cronbach’s alpha of 0.79, and the ACE questionnaire had a Cronbach’s alpha of 0.71.

## Results

**Figure 1:**
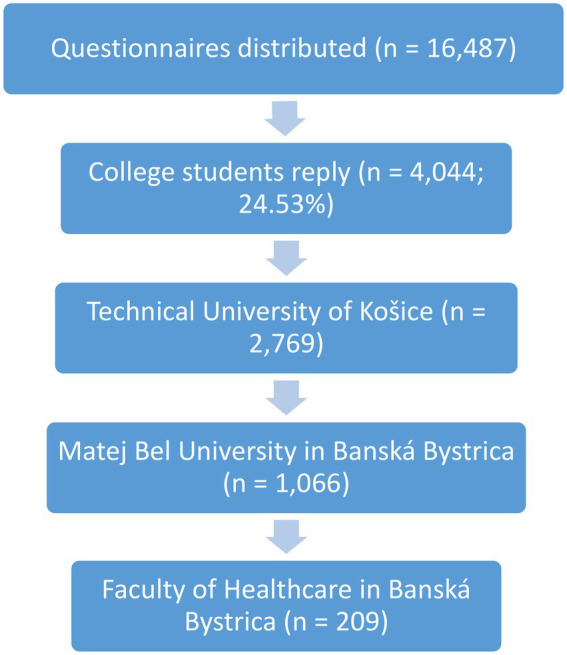
Flow diagram of the study.

A total of 16,487 students received the questionnaire link. The total number of students who answered the questionnaire and were enrolled in the study was 4,044 (24.53%), which included 1,850 women (45.7%) and 2,194 men (54.3%) (see Figure 1).

The AUDIT was completed by a total of 3,647 students. Low AUDIT scores (0–7 points) were detected in 2,573 student participants (1,318 men, 1,258 women), medium scores (8–15 points) in 893 student participants (583 men, 310 women), high scores (16–19 points) in 92 student participants (68 men, 24 women), and very high scores (20–40 points) in 86 student participants (61 men, 25 women). The results are summarized in Table 2.

**Table 2:** Summary results of the AUDIT questionnaire according to the particular scores, domains, and genders.

Domains	Item of the AUDIT	Gender	Particular AUDIT score				
			(0)	(1)	(2)	(3)	(4)
Hazardous alcohol use	Q1. How often do you have a drink containing alcohol?	Women	4.5%	43.4%	24%	23.4%	4.8%
		Men	6.5%	34.5%	23%	24%	12%
		*All*	*5.6%*	*38.6%*	*23%*	*24%*	*8.8%*
	Q2. How many drinks containing alcohol do you have on a typical day when you are drinking?	Women	43.2%	40.6%	12.2%	3.4%	0.5%
		Men	23.6%	42.5%	22.4%	9.6%	1.8%
		*All*	*32.6%*	*41.6%*	*17.7%*	*6.8%*	*1.2%*
	Q3. How often do you have six or more drinks on one occasion?	Women	52.3%	30.9%	10.2%	4.3%	2.2%
		Men	33.7%	27.3%	16.1%	10.5%	12.5%
		*All*	*42.2%*	*29.0%*	*13.4%*	*7.7%*	*7.8%*
Dependence symptoms	Q4. How often during the last year have you found that you were not able to stop drinking once you had started?	Women	87.8%	8.2%	2.4%	0.7%	1.0%
		Men	86.9%	6.9%	3.6%	1.2%	1.4%
		*All*	*87.3%*	*7.5%*	*3.0%*	*1.0%*	*1.2%*
	Q5. How often during the last year have you failed to do what was normally expected from you because of drinking?	Women	82.4%	14.1%	2.3%	0.9%	0.4%
		Men	79.2%	14.5%	4.3%	1.3%	0.7%
		*All*	*80.7%*	*14.3%*	*3.4%*	*1.1%*	*0.5%*
	Q6. How often during the last year have you needed a first drink in the morning to get yourself going after a heavy drinking session?	Women	97.2%	1.7%	0.5%	0.2%	0.3%
		Men	93.0%	4.7%	1.0%	0.6%	0.7%
		*All*	*95.0%*	*3.3%*	*0.8%*	*0.4%*	*0.5%*
Harmful alcohol use	Q7. How often during the last year have you had a feeling of guilt or remorse after drinking?	Women	72.4%	20.9%	5.0%	0.9%	0.8%
		Men	72.2%	21.5%	4.5%	1.1%	0.8%
		*All*	*72.3%*	*21.2%*	*4.7%*	*1.0%*	*0.8%*
	Q8. How often during the last year have you been unable to remember what happened the night before because you had been drinking?	Women	75.7%	19.4%	3.6%	0.7%	0.6%
		Men	67.2%	24.1%	6.5%	1.6%	0.6%
		*All*	*71.1%*	*21.9%*	*5.2%*	*1.2%*	*0.6%*
	Q9. Have you or someone else been injured as a result of your drinking?	Women	82.6%		11.4%		6.0%
		Men	78.0%		15.1%		6.9%
		*All*	*80.1%*		*13.4%*		*6.5%*
	Q10. Has a relative or friend or a doctor or another health worker been concerned about your drinking or suggested you cut down?	Women	95.8%		2.0%		2.2%
		Men	92.3%		3.2%		4.5%
		*All*	*93.9%*		*2.7%*		*3.5%*

Particular score explanation: item Q1: 0 (never), 1 (monthly or less), 2 (2–4 times a month), 3 (2–3 times a week), or 4 (4 or more times a week); item Q2: 0 (1–2), 1 (3–4), 2 (5–6), 3 (7–9), or 4 (10 or more); items Q3–Q8: 0 (never), 1 (less than monthly), 2 (monthly), 3 (weekly), or 4 (daily or almost daily); items Q9–Q10: 0 (no), 2 (yes, but not in the last year), or 4 (yes, during the last year).

Compared to men, the incidence of emotional abuse by a parent, physical abuse by a parent, and sexual abuse by anyone was significantly higher in women (*p* < 0.001). Similarly, women reported greater emotional and physical neglect, growing up with an alcohol and/or drug abuser at home, and living with a family member experiencing mental illness (p < 0.001). The results are summarized in Table 3.

**Table 3:** ACE results according to the type and gender.

Domain of the ACE	Gender	Proportion of ´YEŚ	*p*-value^*^	Frequencies	
				**´YEŚ**	**´NO´**
1. Emotional abuse by a parent	Women	19.8%	<0.001	367	1,483
	Men	11.5%		252	1,942
	*All*	**15.3%**		*619*	*3,425*
2. Physical abuse by a parent	Women	12.2%	<0.001	225	1,625
	Men	8.2%		181	2,013
	*All*	**10.0%**		*406*	*3,638*
3. Sexual abuse by anyone	Women	16.2%	<0.001	300	1,550
	Men	2.9%		63	2,131
	*All*	**8.9%**		*363*	*3,681*
4. Emotional neglect	Women	21.9%	<0.001	406	1,444
	Men	10.5%		230	1,964
	*All*	**15.7%**		*636*	*3,408*
5. Physical neglect	Women	9.9%	<0.001	184	1,666
	Men	5.8%		128	2,066
	*All*	**7.7%**		*312*	*3,732*
6. Parental separation/divorce	Women	23.2%	<0.001	429	1,421
	Men	18.2%		399	1,795
	*All*	**20.5%**		*828*	*3,216*
7. Battered mother	Women	17.0%	<0.001	*315*	*1,535*
	Men	13.5%		*297*	*1,897*
	*All*	**15.1%**		*612*	*3,432*
8. Growing up with an alcohol and/or drug abuser in the household	Women	20.1%	<0.001	372	1,478
	Men	12.9%		284	1,910
	*All*	**16.2%**		*656*	*3,388*
9. Living with a family member experiencing mental illness	Women	20.1%	<0.001	372	1,478
	Men	12.5%		274	1,920
	*All*	**15.9%**		*646*	*3,398*
10. Experiencing the incarceration of a household member	Women	7.2%	0.0348	134	1,716
	Men	5.8%		128	2,066
	*All*	**6.5%**		*262*	*3,782*

^*^According to two-proportion *z*-test, pooled for H0: *p*1 = *p*2.

**Figure 2:**
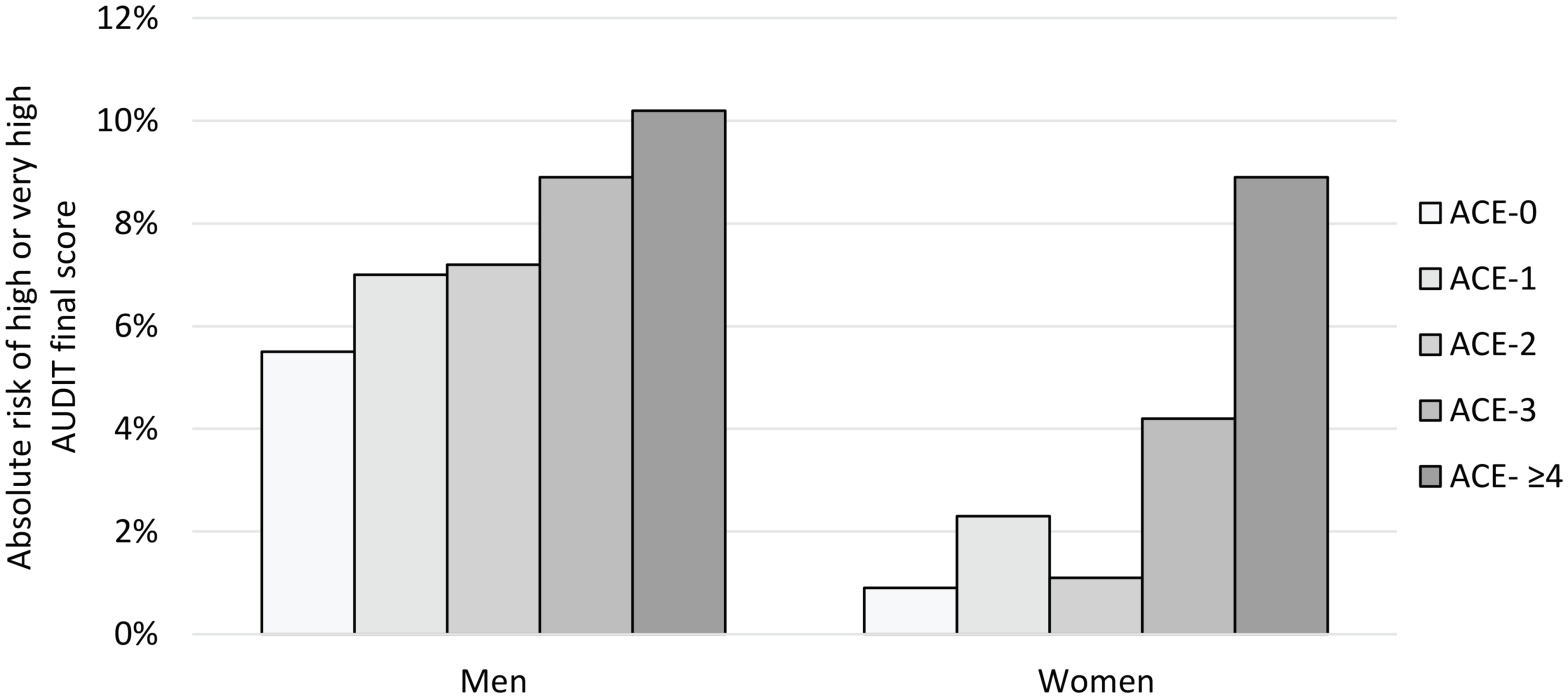
Absolute risk of high or very high AUDIT score in relation to ACE score, separately for men and women.

The incidence of a high or very high AUDIT score in college students with ACE-0, ACE-1, ACE-2, ACE-3, and ACE-4+ was 3.8, 4.7, 4.1, 6.4, and 9.3%, respectively (*p* < 0.001 for ACE-0 vs. ACE-4+). Students who scored an AUDIT score suggesting an increased risk of developing AUD (16–40 score) also scored higher in ACE. These associations are seen in Figure 2.

Of the men who had high or very high AUDIT scores, 5.5% reported no ACE and 10.2% reported an ACE score of 4 or more (*Z* = 2.14; *p* = 0.016). Of women who had high or very high AUDIT scores, 0.9% reported having no ACEs and 8.9% reported an ACE score of 4 or more (*Z* = 6.19; *p* < 0.001) (see Table 4).

**Table 4:** Distribution of participants according to ACE score and AUDIT final score.

ACE score	AUDIT final score			
	Low	Medium	High or very high	Total
0	73.6%	22.6%	3.8%	100%
1	71.3%	23.9%	4.7%	100%
2	67.7%	28.1%	4.1%	100%
3	67.0%	26.7%	6.3%	100%
≥4	63.2%	27.6%	9.3%	100%
Total	70.6%	24.5%	4.9%	100%

For students who had an ACE of 0–3 and a final AUDIT score in the high range, a statistically significant difference was found between men and women, with men being more prone to association between ACE and AUDIT than women (*Z* = 6.41; *p* < 0.001). For students with ACE scores of at least 4 and a high AUDIT final score, no statistically significant differences were found between men and women (*Z* = 0.44; *p* = 0.663; Figure 2).

Comparison of the mean score between the group of alcohol users and the group of non-alcoholics showed that there was a statistically significant difference between these groups (*Z* = 4.559, *p* < 0.001) (Table 5).

**Table 5:** Groups of alcohol users and non-users - descriptive statistics of ACE score.

Group	*N*	Mean ACE score	*SD*	*Z* *	*p*-value
Alcohol users	1,071 (29.4%)	1.53	1.9	4.559	< 0.001
Alcohol non-users	2,576 (70.6%)	1.23	1.7		

* According to Mann–Whitney *U* test.

## Discussion

Slovakia belongs to countries with high alcohol consumption (Chapman et al., 2004; Ford et al., 2011). In our study, an association was observed between a higher incidence of ACEs and alcohol consumption among college students. More ACEs were associated with increased alcohol consumption in both male and female university students. The higher incidence of ACEs may represent one of the main aspects of the development of excessive alcohol consumption in later life. In our study, a high prevalence of ACEs was found; ACEs were not rare and were more common in women than in men. In college students, a low proportion of abstinent students was found. College students in their twenties are less than 6% absolute abstainers (together with the alarming 8.8% of frequent and binge drinkers). Slovakia ranks first in the world in the prevalence of liver cirrhosis, and the mortality of AUD is 1.5 times higher than in the rest of Europe (Sepanlou et al., 2020). These results are very likely reflections of a permissive national alcohol policy. The relationship between increased alcohol consumption was found in both men and women; however, in female students, the incidence of ACEs and their relative impact on the increase in AUDIT was higher compared to male students. It is common knowledge that girls are more likely to be exposed to sexual violence than boys, which is correlated with the results of our study (Finkelhor, 1994). On the other hand, a higher baseline alcohol consumption was found (higher AUDIT scores in students without ACEs) in male students compared to female students. The relative increase in AUDIT scores based on the increase in ACE scores was more pronounced in female students (approximately tenfold increase between ACE-0 and ACE-4+) compared to male students (approximately twofold increase); however, the cumulative proportion of drinkers in any ACE category was higher in male students. Our results show that in male students, additional driving forces behind alcohol consumption, in addition to ACEs, are important.

The links between gender, early ACEs, and self-reported binge drinking and heavy drinking were studied by Crouch et al. (2018). Almost all ACE categories were associated with an increased probability of reporting binge drinking and heavy drinking. Mental illness had the highest odds for men, and emotional abuse had the highest odds for women. Men and women with four or more ACEs had a higher chance of reporting binge and heavy drinking compared to their counterparts. In addition to these findings, our results point to the difference in the strength of impact ACEs can exert on drinking of women compared to men (10-fold increase compared to a 2-fold increase).

ACEs are associated with a higher risk of anxiety and depression in old age (Chapman et al., 2004; Ford et al., 2011; Hovens et al., 2015). A better understanding of the factors underlying the risk of problem drinking and dependence on alcohol is important for the development of better prevention and early intervention measures. Depression among adult children of alcoholics appears to be largely, if not exclusively, associated with an unfavourable family experience with their parents (Anda et al., 2002). ACEs are common and strongly associated with subsequent alcohol abuse and are likely to account for a high proportion of alcohol abuse (Leung et al., 2016). Children in alcoholic families have more frequent adverse experiences (Wolock and Magura, 1996); however, the risk of alcoholism and depression in adulthood increases with the increasing number of ACEs, regardless of parental alcohol abuse (Wolock and Magura, 1996). The effects of parental substance use (including alcohol) on abuse outcome of their children’s appear to be partly mediated by their neglectful parenting (Rothman et al., 2008; Pilowsky et al., 2009). Loudermilk et al. (2018) studied the association between binge drinking and ACEs. Adults who experienced household abuse were 30% more likely to drink. As in our study, men were at increased risk of becoming alcoholics. The association between alcohol consumption and ACEs was also studied by Koss et al. (2003). There were significant sex differences in alcohol dependence and several ACEs. High prevalence rates for one or more types of ACEs were documented (men: 74–100%; women: 83–93%). For men, combined physical and sexual abuse significantly increased the likelihood of subsequent alcohol dependence; for women, sexual abuse, and attendance at boarding schools increased the odds of alcohol dependence. In a study by Batty et al. (2008), 8,170 individuals born in 1970 were tested for alcohol drinking. Higher childhood mental ability scores had an increased prevalence of problem drinking in adulthood. Unlike our study, this association was stronger in women. Childhood mental ability was also associated with a higher average intake of alcohol and a higher frequency of drinking.

Our study has several limitations. The main limitation of our study is the retrospective nature of the study, which is associated with several risks of bias. It is difficult to determine a direct association according to cross-sectional analysis. Therefore, it is difficult to make clear recommendations. Study was done during the pandemic COVID-19 which may have influenced the outcome of alcohol consumption.

The questionnaires were distributed through the schools’ online communication systems, and their completion was voluntary. This could have led to a selection bias. The veracity of obtained data on the ACE test and the AUDIT cannot be proved, and therefore, some answers may be underestimated or overestimated. Finally, since the level of education also depends on the ACE score, the results of our study may not reflect other segments of the population. On the other hand, the strengths of the study are its size of several 1000’s and the rarity of alcohol consumption that is analysed against ACE in the regional and social environment of college students.

## Conclusion

Among university students, ACEs were not rare and were more frequent in women than in men. Higher ACE scores were associated with higher AUDIT scores in both men and women. Men without ACEs have higher AUDIT scores than women without ACEs. An association between ACE and AUDIT scores was stronger in female students compared to male students. This points to the gender-specific pathogenesis of alcohol use and AUD, with the underlying driving forces being therapeutic targets.

## Data availability statement

The original contributions presented in the study are included in the article/Supplementary material, further inquiries can be directed to the corresponding author.

## Ethical statement

The studies involving human participants were reviewed and approved by Ethical Committee of Matej Bel University. The patients/participants provided their written informed consent to participate in this study.

## Author contributions

KŠ, ĽS, EL, and MM: conceptualization. ĽS, DL, TK, and KŠ: methodology. ĽS: validation and supervision. ĽS, KŠ, MS, and MM: formal analysis. DL, EL, and KŠ: investigation. KŠ, DL, ĽS, MP, and MM: writing. KŠ, DL, and EL: original draft preparation. KŠ, DL, EL, MS, MP, TK, LB, MM, and ĽS: writing—review and editing. EL and MS: project administration. All authors contributed to the article and approved the submitted version.

## Funding

This research was funded by the Ministry of Health, Czech Republic; conceptual development of research organization (FNBr, 65269705).

## Conflict of interest

The authors declare that the research was conducted in the absence of any commercial or financial relationships that could be construed as a potential conflict of interest.

## Publisher’s note

All claims expressed in this article are solely those of the authors and do not necessarily represent those of their affiliated organizations, or those of the publisher, the editors and the reviewers. Any product that may be evaluated in this article, or claim that may be made by its manufacturer, is not guaranteed or endorsed by the publisher.

## Supplementary material

The Supplementary material for this article can be found online at: https://www.frontiersin.org/articles/10.3389/fpsyg.2022.1004651/full#supplementary-material
