# Supplementary material for: Relationship between alcohol consumption and adverse childhood experiences in college students–A cross-sectional study
Source: Front Psychol. 2022 Oct 12;13:1004651. doi: 10.3389/fpsyg.2022.1004651 (PMC9605734; doi:10.3389/fpsyg.2022.1004651)
Supplement: Supplementary file 1 [file Table_1.DOCX]

Finding Your ACE Score

While you were growing up, during your first 18 years of life:

1. Did a parent or other adult in the household often or very often… Swear at you, insult you, put you down, or humiliate you? or

Act in a way that made you afraid that you might be physically hurt?

Yes No If yes enter 1 ________

1. Did a parent or other adult in the household often or very often… Push, grab, slap, or throw something at you? or

Ever hit you so hard that you had marks or were injured?

Yes No If yes enter 1 ________

1. Did an adult or person at least 5 years older than you ever…

Touch or fondle you or have you touch their body in a sexual way? or

Attempt or actually have oral, anal, or vaginal intercourse with you?

Yes No If yes enter 1 ________

1. Did you often or very often feel that …

No one in your family loved you or thought you were important or special? or

Your family didn’t look out for each other, feel close to each other, or support each other?

Yes No If yes enter 1 ________

1. Did you often or very often feel that …

You didn’t have enough to eat, had to wear dirty clothes, and had no one to protect you? or

Your parents were too drunk or high to take care of you or take you to the doctor if you needed it?

Yes No If yes enter 1 ________

1. Were your parents ever separated or divorced?

Yes No If yes enter 1 ________

1. Was your mother or stepmother:

Often or very often pushed, grabbed, slapped, or had something thrown at her? or

Sometimes, often, or very often kicked, bitten, hit with a fist, or hit with something hard? or

Ever repeatedly hit at least a few minutes or threatened with a gun or knife?

Yes No If yes enter 1 ________

1. Did you live with anyone who was a problem drinker or alcoholic or who used street drugs?

Yes No If yes enter 1 ________

1. Was a household member depressed or mentally ill, or did a household member attempt suicide?

Yes No If yes enter 1 ________

1. Did a household member go to prison?

Yes No If yes enter 1 _______

Now add up your “Yes” answers: _______ This is your ACE Score.
